# New classification of endometrial cancers: the development and potential applications of genomic-based classification in research and clinical care

**DOI:** 10.1186/s40661-016-0035-4

**Published:** 2016-12-13

**Authors:** A. Talhouk, J. N. McAlpine

**Affiliations:** 1 Department of Pathology and Laboratory Medicine, University of British Columbia and BC Cancer Agency, Vancouver, BC Canada; 2 Department of Gynecology and Obstetrics, Division of Gynecologic Oncology, University of British Columbia, 2775 Laurel St. 6th Floor, Vancouver, BC Canada V5Z 1M9

**Keywords:** Endometrial carcinoma, Histotype, The Cancer Genome Atlas (TCGA), Risk stratification, Prognosis, *POLE* mutations, Mismatch repair deficiencies, p53, Molecular classification

## Abstract

Endometrial carcinoma (EC) is the fourth most common cancer in women in the developed world. Classification of ECs by histomorphologic criteria has limited reproducibility and better tools are needed to distinguish these tumors and enable a subtype-specific approach to research and clinical care. Based on the Cancer Genome Atlas, two research teams have developed pragmatic molecular classifiers that identify four prognostically distinct molecular subgroups. These methods can be applied to diagnostic specimens (e.g., endometrial biopsy) with the potential to completely change the current risk stratification systems and enable earlier informed decision making. The evolution of genomic classification in ECs is shared herein, as well as potential applications and discussion of the essential research still needed in order to optimally integrate molecular classification in to current standard of care.

## Background

Cancer care in the last decade has featured a concerted move towards the personalization of patient care, often called precision medicine. In the field of cancer, this has meant a progression from broad categorization of tumors by anatomic site, to distinguishing subgroups by histomorphology, and more recently defining tumors by molecular features. This evolution has not happened over night and pace of change has varied by tumor site. Paradoxically, despite endometrial cancer being the most common gynecologic malignancy in women in Canada and the United States [1, 2] and the 6^th^ most common cancer in women globally [3], research and clinical advancement have arguably lagged as compared to other cancers. This may be because over 75% of women diagnosed with endometrial cancer have early stage disease (stage I or II) and favorable outcomes (5-year overall survival 75–90%) [4–6]. However, for those women who recur or for those who present with more advanced disease, response rates to conventional chemotherapy are low and clinical outcomes are extremely poor [7–10].

Renewed research focus on this disease site has been prompted by a dramatic increase in incidence observed in the developed world [2, 11–13]. In addition, there has been frustration with contemporary practice, in part due to inconsistent EC histomorphologic categorization, imprecise risk stratification, and diverse treatment strategies. Multidisciplinary panel recommendations on management of ECs [14] have emerged in an effort to make treatment (surgery, chemotherapy, radiotherapy, surveillance) more consistent. Multiple reviews on state of the art care of EC’s have been published, and increasingly the repercussions of treatment on patient quality of life are being assessed in addition to survival parameters [6, 15–20]. Attention to this balance of treatment and sequelae may be even more essential in this disease site as there is concern that many women are likely over-treated or under-treated.

There has been a call for the incorporation of molecular features in to both classification and risk determination of ECs in order to better assess the biological behavior of an individual’s disease and ultimately to improve treatment decisions and outcomes [21, 22]. The objective of this review is to focus on the new genomic framework used to categorize endometrial carcinomas. Herein we describe the evolution of molecular classification systems and how genomic characterization will impact both research approach and clinical management for this disease.

## Historical/Pathogenetic Classification of Endometrial Cancer

Thirty years ago, Bokhman hypothesized there were two pathogenetic types of endometrial carcinomas driven by very different metabolic and endocrine signals [23]. Type 1 is more common (~70–80%), consisting of endometrioid, low grade, diploid, hormone-receptor positive tumors that are moderately- or well-differentiated and more common in obese women. Patients presenting with Type 1 tumors tend to have localized disease confined to the uterus and a favourable prognosis. In contrast, Type 2 tumors (20–30%) are more common in non-obese women, of non-endometrioid histology, high-grade, aneuploid, poorly differentiated, hormone receptor negative and associated with higher risk of metastasis and poor prognosis. While this historical system of taxonomy has been useful, substantial heterogeneity within and overlap between Type I and II cancers is now recognized. Type I and Type II designation has never been part of the formal staging nor risk stratification, and thus has no clinical utility beyond providing a conceptual framework for understanding endometrial cancer pathogenesis.

## Endometrial classification by histomorphology and current systems of risk stratification

Tumor grade and histologic subtype assessment are subjectively assigned according to appearance under the microscope and predefined pathologic criteria. Nuclear features and the proportion of solid tumor vs. identifiable glands defines grade 1–3. Histologic subtype is assigned by morphologic criteria and often aided by immunostains. Pathologic accuracy is hampered by poor diagnostic reproducibility, especially in the case of high-grade subtypes (e.g. grade 3 endometrioid, serous). Studies describe inter-observer disagreement or lack of consensus on histologic subtype diagnosis in one-third or higher of ECs [24–27]. The overall kappa statistics for FIGO grade assignment between pathologists is 0.41–0.68, indicative of only moderate levels of inter-observer agreement [24, 28]. Agreement between diagnostic specimens and final hysterectomy is also limited [29–32]. In short, histologic classification is not accurate or precise enough to effectively triage patients into optimal treatment groups.

Endometrial carcinoma has been a surgically staged disease since 1988. Surgery traditionally involves hysterectomy with bilateral salpingo-oophorectomy +/− lymph node dissection or sampling and omentectomy with several safe options in surgical approach [14, 33–35]. Extent of staging may vary according to patient age, comorbidities, cancer histology, grade, disease distribution, surgeon preference and institutional practice. Surgery alone is typically sufficient to cure early-stage EC [14, 36, 37], however, it is recognized that tumors with ‘high-risk’ features have a high likelihood of recurrence and adjuvant treatment (radiation and/or chemotherapy) is recommended [8, 16, 38, 39]. The major challenge is in distinguishing the features that comprise ‘low-‘, ‘intermediate-‘, and ‘high-risk’ disease in ECs. Multiple different risk predictive clinical models have been developed to guide treatment [14, 37, 40–48]. These have evolved with new FIGO staging and through interpretation of large clinical trials, however all incorporate the key pathological parameters of histotype, grade, and stage. As mentioned previously, the reproducibility of both histotype and grade have been demonstrated to be poor in EC’s [24, 26, 27], thus two of three major criteria for risk group assignment which directly impacts recommendations for adjuvant treatment have limited reproducibility. Understandably, this makes it challenging to confidently make treatment decisions. We know that some women are undertreated who could have benefited from aggressive surgery, chemotherapy and/or radiation, and many may be overtreated having been cured by surgery alone.

The adequacy of risk stratification systems in EC have recently been compared and challenged [22, 49]. There are five major risk stratification systems in EC, of which the modified European Society of Medical Oncologists (ESMO) classification was demonstrated to best discriminate for recurrence and nodal metastases in apparent early stage disease [49]. However, none of the existing schemes were deemed highly accurate. In addition, all current systems stratify women based on pathologic data obtained *after* surgical staging (stage is a component of risk assignment). There is great need to obtain *earlier* and more *biologically informative* data from EC tumors that could assist in planning the optimal course of treatment for the individual. In addition, diagnostic tools that could objectively and consistently categorize ECs into distinct subgroups would enable stratification of clinical trials and study of treatment efficacy within biologically ‘like’ subgroups. Stemming from clinical need and a recognized inadequate/unsustainable system a call was made for the integration of molecular features.

## A new genomic era: molecular classification of endometrial carcinomas

Several research teams have defined immunohistochemical and/or mutation profiles to aid in distinguishing EC subtypes [50–58]. In one series, a set of seven immunohistochemical markers was able to improve the distinction between high-grade EC histotypes [28] and more recently, another team demonstrated a nine protein panel improved identification of both low and high-grade EC subtypes [57]. Sequencing has enabled further improvement, with a nine-gene panel, demonstrating distinct mutational profiles for the major EC histotypes [52]. Molecular data has also been used to further stratify risk categories; using gene expression profiling and copy number analysis to determine risk of recurrence [59, 60], even in apparent low stage disease [61]. Molecular characterization has also been pursued for potential therapeutic targets in EC, focusing on frequently mutated pathways such as PI3K/PTEN/AKT/mTOR. Further work is needed to define molecular biomarkers that more accurately reflect tumor susceptibility [62–66].

The most comprehensive molecular study of ECs to date has been The Cancer Genome Atlas (TCGA) project, which included a combination of whole genome sequencing, exome sequencing, microsatellite instability (MSI) assays, and copy number analysis [67]. Molecular information was used to classify 232 endometrioid and serous endometrial cancers into four groups - *POLE* ultramutated, MSI hypermutated, copy-number (CN) low, and CN high - that correlate with progression-free survival.

The ultramutated *POLE* subgroup was a novel finding from the TCGA, and generated interest due to its very favorable outcomes even within high-grade tumors. In TCGA, ultramutated cases were characterized by *POLE* exonuclease domain mutations (EDM), a high percent of C > A transversions, a low percent of C > G transversions, as well as more than 500 SNVs. *POLE* encodes the major catalytic and proofreading subunits of the Polε (Polymerase Epsilon) DNA polymerase enzyme complex responsible for leading strand DNA replication. The exonuclease proofreading function and the high fidelity incorporation of bases by *POLE* ensures a low mutation rate in the daughter strand. In ECs, *POLE* EDMs are mostly found in hotspot regions with V411L and P286R being the most common mutations. Substitutions in DNA polymerases were shown to inactivate or suppress proofreading abilities, thus causing increased replicative error rates and resulting in the ultra-mutated phenotype. In the TCGA, whole genome or exome sequencing was used to assess *POLE* status. Other series have subsequently assessed *POLE* status using more focused methods including Sanger sequencing [68, 69], gene panels [69–71], digital PCR [72–74] or functional assays [75] and confirmed very favourable outcomes for women with *POLE* aberrant ECs.

TCGA also described a molecular subgroup that exhibited microsatellite instability (MSI). MSI arises from defects in post-replicative DNA mismatch repair system. In the TCGA, MSI was determined by a panel of four mononucleotide repeat loci (polyadenine tracts BAT25, BAT26, BAT40, and transforming growth factor receptor type II) and three dinucleotide repeat loci (CA repeats in D2S123, D5S346, & D17S250) in addition to the recommended markers from the National Cancer Institute [76], tumor DNA was classified as microsatellite- stable (MSS) if zero markers were altered, low level MSI (MSI-L) if one to two markers (less than 40%) were altered and high level MSI (MSI-H) if three or more markers (greater than 40%) were altered. Mismatch repair deficiencies can result from i) an inherited cancer syndrome (e.g., Lynch), ii) acquired/somatic mutations or iii) epigenetic events e.g. methylation of one of the genes involved in mismatch DNA repair, most commonly MLH1.

Finally TCGA distinguished a distinct molecular subgroup by copy number analysis. Copy number was determined using Affymetrix SNP 6.0 microarrays using DNA originating from frozen tissue. Hierarchical clustering identified significantly reoccurring amplifications or deletions regions and a ‘copy number (CN) high’ subgroup. All remaining samples that did not belong to the *POLE* ultramutated group, the MSI group, or the CN high group, were termed CN low. The appeal of objective molecular categorization of new EC cases in to one of four prognostic subgroups was immediately apparent. However, methodologies used for the TCGA study were costly, complex and unsuitable for wider clinical application.

**Fig. 1:**
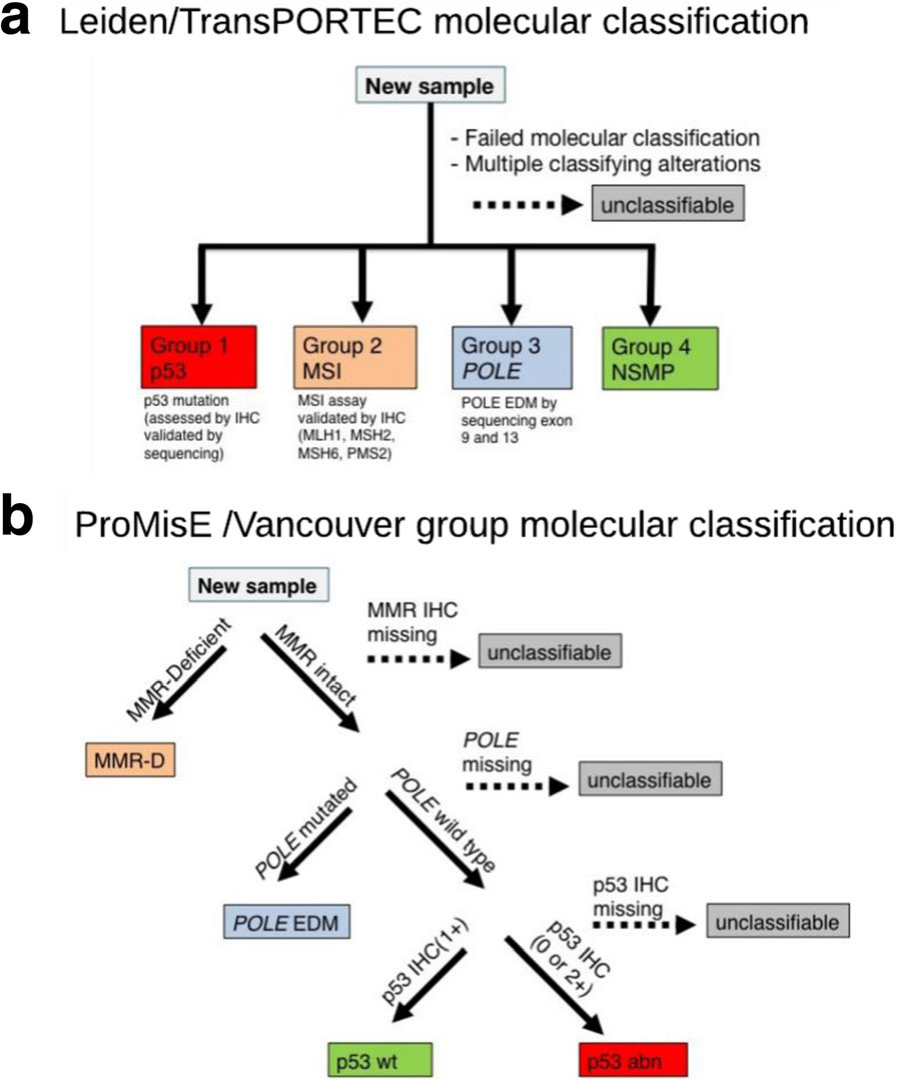
Schematic of the **a** Leiden/TransPORTEC and **b** ProMisE/Vancouver molecular classification systems including testing performed, molecular subgroups identified, and by what criteria cases would be considered unclassifiable

Two research teams, including our own, have subsequently developed more pragmatic methodologies to evaluate molecular features of ECs, working in standard formalin-fixed paraffin-embedded tissue. These methods do not identify molecular subgroups that are identical to TCGA but do recapitulate the four survival curves observed in TCGA [69, 71, 73, 77] (Fig. 1). Stelloo et al. [69, 71] used a combination of *TP53* mutational testing and p53 IHC to determine p53 status obtained from sequencing as a surrogate for CN high TCGA subgroup. The promega MSI analysis system was used to determine MSI status. For tumors exhibiting low levels of instability or from which extracted DNA quality was poor, immunohistochemistry for mismatch repair (MMR) proteins (MLH1, MSH2, MSH6, and PMS2) was performed. *POLE* EDM hotspot mutations were identified by Sanger sequencing. This team also tested for hotspot mutations (159) across 13 genes (*BRAF, CDKNA2, CTNNB1, FBXW7, FGFR2, FGFR3, FOXL2, HRAS, KRAS, NRAS, PIK3CA, PPP2R1A,* and *PTEN*). Testing ultimately yielded four molecular subgroups: group 1 - p53 (mutation identified), group 2- MSI, group 3 –*POLE* (*POLE* EDM identified), and finally group 4 –NSMP, a group with ‘no specific molecular profile’ (Fig. 1a). Tumors with insufficient tissue to perform all molecular testing were not classified and tumors with more than one molecular feature, constituting 2–3% of the cohort, were also not classified. Due to this exclusion, the order of mutational testing was irrelevant. This research team initially assessed ECs from the PORTEC3 trial (*n* = 116), with known high risk features. Recurrence-free survival and time to distance metastasis were assessed within the four molecular subgroups. They observed that patients belonging to the *POLE* and the MSI subgroups showed similar and much better survival outcomes in comparison to the p53 mutant group and the NSMP group which exhibited worse recurrence and distance metastasis outcomes even within the endometrioid histology cases. Differences in survival patterns relative to the TCGA results were attributed to a greater proportion of high-risk features in the PORTEC 3 cohort.

The Leiden/TransPORTEC group has since applied the same series of molecular tests to a larger, more diverse cohort [71]. However, survival analysis and assessment of prognostic ability was restricted to endometrioid subtype and stage 1 tumors of patients with intermediate clinical risk. Within this very specific group, the observed outcomes associated with each molecular subgroup more closely mirrored TCGA.

Our research team has also developed a molecular classification system that uses practical methodologies to assign ECs to one of four molecular subgroups with distinct survival outcomes. We have followed the Institute of Medicine (IOM) guidelines for the development of ‘omics based tests [78], initially exploring 16 models in a ‘discovery’ cohort (*n* = 141) [73], next locking down sequence of testing and methods to a single model termed ProMisE (Proactive Molecular Risk Classifier for Endometrial Cancer) on a new ‘confirmation’ cohort (*n* = 319) [77, 79] to prove feasibility and confirm the association with outcomes/prognosis, and finally testing in a large ‘validation’ cohort (*n* = ~500) of ECs from collaborators at the University of Tübingen (Germany). Molecular decision tree analysis for ProMisE is outlined in Fig. 1b. Specific methodologies include immunohistochemistry (IHC) for the detection of the presence/absence of two mismatch repair (MMR) proteins: MSH6 and PMS2. This identifies ‘MMR-D’ (deficient) subgroup. Cases are then sequenced using digital PCR to identify *POLE* exonuclease domain mutations (‘*POLE* EDM’). Finally, cases are assessed using IHC for p53 (wild type vs. null or missense mutations; ‘p53wt’ and ‘p53abn’, respectively). We have demonstrated that women within each molecular subgroup have clinicopathological characteristic that have consistently been shown to be typical of that group. For example, the p53 abn subgroup usually encompasses the highest proportion of high grade, advanced stage, non-endometrioid histotypes and arises in older, thinner women. Similarly, the emerging phenotype of women whose EC harbor *POLE* EDMs is of particular interest since it generally includes younger, thinner and with surprisingly aggressive pathologic features (large proportion of grade 3 tumors, many with deep myometrial invasion and LVSI) yet consistently exhibit favorable outcomes. The MMR-D subgroup have very similar ‘uterine factors’ (clinicopathologic features in the uterus itself) [48] to the *POLE* subgroup, i.e. a comparable proportion of high grade tumors and deep myometrial invasion and LVSI, yet they have worst observed outcomes of any group next to p53abn [77, 79]. On multivariable analysis, ProMisE molecular subgroup assignment maintained its association with overall survival (OS), progression free survival (PFS) and recurrence free survival (RFS) even after correction of other clinicopathologic parameters of known prognostic significance available at time of diagnosis/collection of diagnostic specimen for molecular analysis (e.g., age, BMI, grade, histotype but not stage).

Both ProMisE (across all tumors tested), and the Leiden classifier (within the intermediate-risk group examined) demonstrate comparable risk discriminatory ability to the ESMO risk stratification system. Furthermore when clinical and pathological features were integrated with molecular features they resulted in improved risk stratification. Through evaluation of the collective cohort (discovery + confirmation + validation cohorts = ~ 1000 ECs) we plan to evaluate which key clinicopathological parameters can add value to molecular classification giving high priority to those features available at time of diagnosis (e.g., age, BMI).

Our goal has consistently been to develop a molecular classification tool that could be applied to diagnostic specimens (endometrial biopsy or curettage) and therefore inform treatment at the earliest time point. Biologically relevant information about an individual’s tumor could guide surgical urgency and aggressiveness, fertility or hormonal function sparing management options, adjuvant therapy, and/or surveillance schedules. We have demonstrated high concordance between ProMisE molecular classification in diagnostic vs. final hysterectomy samples, far superseding concordance of grade, or histotype as assigned on original pathology reports or within or between reviews by expert gynecologic cancer pathologists [80]. The Leiden team has also shown high concordance of molecular tumor alterations between pre-operative curettage specimens and final hysterectomy specimens (13 gene panel and MSI assay) [81] and a multicenter, prospective trial in Holland is in process to see if surgical management can be improved [82]. As diagnostic specimens are fixed immediately (in contrast to a hysterectomy specimen that may sit for hours in an operating room before processing in pathology), the quality of DNA extracted and fixation for IHC is high. We believe one of the most exciting aspects of molecular classification and what will be most impactful in directing care for women with EC will be this capability of determining earlier prognostic (and possibly predictive) information.

Ultimately, integration of molecular classification by either method into current practice, as performed on diagnostic specimens or final hysterectomy, will need to be studied in the context of a prospective clinical trial; comparing survival outcomes, quality of life and health economic implications to conventional/historical standard of care.

## Challenges with molecular classification: key components

The Leiden/TransPORTEC and Vancouver/ProMisE pragmatic molecular classification systems incorporate the same integral components: identification of ECs with mismatch repair deficiency/microsatellite instability, *POLE* exonuclease domain mutations and aberrant p53. Similarities and differences are shown in Fig. 1. Prognostic strength of molecular classification is at least equivalent to other clinicopathological features or risk stratification systems but offers the advantage of objective results (e.g., presence or absence of a protein or mutation). We believe these key molecular components are unlikely to be outperformed by any single clinicopathological parameter or biomarker. Notably, as yet none of the additional immunohistochemical markers we have tested across our endometrial cancer cases have outperformed ProMisE. Although we and others are investigating the immune landscape and specific immunohistochemical biomarkers within the context of these major molecular subgroups these studies will not be covered in this manuscript. Should any parameter improve the ability to discern outcomes and guide management beyond the ProMisE or Leiden molecular classification, they can be incorporated into future algorithms. Herein, we focus on some of the major challenges and considerations for future implementation of molecular classification.

## MMR/MSI

There are different techniques for the identification of mismatch repair deficiency [76, 83–87]. Both TCGA and the Leiden series use microsatellite instability (MSI) assays. These have primarily been utilized in research, not clinical practice settings (there are no FDA-approved MSI tests) and require DNA extraction from tumor as well as normal tissue or blood for comparison. ProMisE tests for the presence of two mismatch repair proteins (MSH6, PMS2) by immunohistochemistry and we have shown high concordance between MMR IHC and MSI assay methods [83]. IHC staining for MMR and interpretation is routine for most pathology laboratories. Unfortunately, although histomorphologic surrogates for MMR deficiency or Lynch syndrome have been explored (e.g., tumor infiltrating and/or peritumoral lymphocytes, dedifferentiated histology, lower uterine segment origin) [88, 89], as yet they have not proven to be equivalent to molecular confirmation.

Although all MMR deficiencies are often grouped together, for inherited mutations (Lynch syndrome), the lifetime risk and age of penetration of Lynch-associated cancers can vary substantially according which gene is aberrant [90]. This may impact recommendations regarding the timing of screening or intervention e.g., lower lifetime risk and later average age of penetration for individuals with aberrant MSH6 [90–93] might enable delay of recommended risk reducing surgery as compared to other Lynch mutations.

Prognostic and predictive implications of mismatch repair may also vary according to specific MMR gene mutation or protein loss identified. It has been hypothesized that epigenetic/methylation events in mismatch repair likely have different implications on tumor characteristics and clinical outcomes than germline defects e.g. an age-related somatic event would not be expected to promote the development of tumor that is equivalent to one arising in a young individual harboring a germline mutation. Immune environment, intrinsic biologic behavior, toleration of adjuvant therapy/response to cell injury may vary significantly in these individuals. This may partially explain the relatively wide range of response to immunotherapy within MMR-D cases. At present, all mismatch repair deficiencies are lumped together but further interrogation of these differences (e.g. subgroups of subgroups) is warranted. Recently, over 1000 women with EC had their tumors evaluated for microsatellite instability, *MLH1* methylation, and MMR protein expression as part of a combined NRG Oncology/Gynecologic Oncology Group Study (GOG210) [94]. Categories of normal mismatch repair, epigenetic defect and probable mutation (somatic or germline) were compared to clinicopathologic variables and clinical outcomes in the trial cohort. Even with this large number of cases, these three broad categories of MMR status were not shown to be associated with PFS or DSS. Univariate analysis did suggest potentially worse PFS for women whose tumors had epigenetic defects (trend, *p* = 0.1) but this association  was not maintained after adjusting for other factors, including the highly relevant parameter of age in this cohort. In addition, the authors observed a trend to improved PFS in tumors with MMR mutations and a suggestion that these patients received greater benefit from adjuvant chemotherapy compared to women with normal mismatch repair. Similar results for probable germline/Lynch syndrome mismatch repair deficient tumors were observed in a smaller series of 221 ECs, with no prognostic nor predictive associations noted in the tumors with methylation events [95].

## POLE

Several research teams have characterized *POLE* mutated tumors by histomorphology and immune environment [96–101]. Obvious clinical implications for tumors with substantial immune infiltrates include selection for anti-PD-1 therapy. However, the highly favorable outcomes observed in women with *POLE* mutated tumors would suggest that costly targeted therapy might better be reserved for the very rare cases of recurrent or advanced disease [102, 103]. *POLE* somatic mutations are found in less than 10% of endometrial carcinomas and recurrence is seldom observed; thus, it has been difficult for a single study to be adequately powered to determine optimal management of women whose tumors harbour this molecular feature. Adjuvant treatment is commonly administered due to the frequency of ‘high-risk’ features in ECs with *POLE* EDMs (e.g., relatively high frequency of grade 3, deep myometrial invasion, LVSI) but whether this is over treatment of women who would do well based on their *POLE* genotype alone or whether treatment is needed and favorable outcomes are secondary to exquisite sensitivity to DNA damaging agents in these tumors is as yet unclear.

The paradox of observed aggressive histopathologic features but excellent survival outcomes may in part be explained by the high neoantigen load and immune rich microenvironment in tumors with *POLE* EDMS (and to a lesser degree, also described in MMR-D tumors).

Both sequencing and functional assays currently employed for *POLE* mutation testing are more costly than IHC and utilize methods that require a skilled team to perform and interpret. We, and others, continue to search for surrogates for *POLE* sequencing. Although the clinical and pathological phenotype of women with *POLE* mutated tumors is beginning to be characterized; on average younger, lower BMI, high proportion of grade 3, LVSI+, predominantly endometrioid, and low stage, these parameters overlap with other molecular subgroups. At present there is no single pathognomonic surrogate for this feature.

## p53

The mutational spectrum of *TP53* mutations within ECs was recently described in Schultheis et al. [104], both in the context of histotype and across TCGA molecular subgroups. This study confirmed the very high proportion (91%) of *TP53* mutations in the ‘CN high’ TCGA category but also seen in 35% of the *POLE* genomic subgroup. No clinical correlative data was provided with their paper but our series and others confirm the highly favorable outcome of POLE mutation carriers even with the identification of other mutations traditionally associated with high risk disease. The order of our categorization: identification and removal of *POLE* subgroup prior to p53 stratification thus seems to be of great importance (see tumors with >1 molecular feature below). Also described in this series was the presence of frameshift or nonsense TP53 mutations (22% of *TP53* mutant subset) of which they acknowledge would yield different IHC results (loss or IHC score 0) than missense variants (IHC score 2). Identification of both aberrant states is essential. Our team, in collaboration with others is in the process of further characterizing both *TP53* mutational and IHC status in ECs in order to better guide interpretation in this disease site.

## Tumors with more than one molecular feature

Both Talhouk et al. [77, 79] and Stelloo [71] et al. describe approximately 2-3% of endometrial tumors having more than one of the key molecular features described. Reported frequency of post-replication *POLE* proofreading defect *and* a DNA mismatch repair defects varies in the literature, but in series where co-occurrence is higher, this has been attributed to somatic MMR mutations which may be secondary to the ultra-mutated *POLE* phenotype. [67, 70, 98]. Similarly, it is perhaps not surprising that in both the *POLE* and MMR-D subgroups of ECs with high mutational loads, tumors may also harbour *TP53* mutations (as evidenced by either sequencing, or complete loss or overexpression of p53 protein on IHC) [71, 77, 79, 104]. The order of testing for molecular classification is therefore critically important. Determination of *POLE* status prior to p53 testing will categorize a given EC as *POLE* EDM. Favorable outcomes are therefore anticipated for that individual, and indeed for cases reported thus far with dual features, that has been observed [77]. We believe testing for MMR-D first is still valid, as that information is arguably more actionable than *POLE* status (referral for hereditary testing, consideration of immunotherapy) which is not currently integrated into treatment algorithms. Ultimately, distinguishing between what are likely passenger mutations or late events without functional consequence as compared to mutations that define biologic behavior is essential. Molecular classification tools that utilize large gene panels may detect a plethora of coexisting mutations in *POLE* EDM ECs and need to be interpreted with caution e.g., discovery of *BRCA1* or *BRCA2* mutation in a *POLE* mutated EC may not indicate homologous recombination deficiency / PARPi efficacy [105].

Clinical outcomes may be harder to discern between ECs demonstrating both MMR deficiency and p53 mutations and the ‘best’ categorization of these tumors remains to be determined. At present, molecular classification will first identify the MMR deficiency at least enabling patients to be referred for hereditary counselling and providing opportunities in genotype specific clinical trials.

## Genotype-phenotype interplay

Genotype-phenotype interactions have been appreciated and characterized in recent years. Although not the focus of this review, we will take this opportunity to describe one highly relevant example.

It is now appreciated that PTEN loss has different prognostic implications in lean vs obese individuals. Mutations in the central relay pathways of insulin signals (phosphatidylinositol 3-kinase (PI3K) pathway including mutations specifically in PIK3CA, PIK3R1 and PTEN) are extremely common in ECs yet prior studies on the prognostic significance of PTEN mutations had markedly discordant results. Westin et al. stratified cases by body mass index (BMI) revealed improved progression free survival in obese (BMI >30) women with endometrioid endometrial carcinoma suggesting an interaction between metabolic state and genetics [106]. Subsequently, a constellation of ‘obesity related’ genes are observed to be upregulated with increasing BMI among endometrioid carcinomas in the TCGA cohort [107], and different targets for treatment were suggested in obese vs non-obese individuals [108]. Given the global epidemic of obesity and associated ‘metabolic syndrome’, this clinical context is essential to know in guiding clinical management and in research/interpretation of data. In our own series, for example, we anticipate, that further stratification of cases within the p53 wt subgroup (and possibly within MMR-D) by BMI status may refine prognosis further. We are in the process of examining the interaction of PTEN and BMI within the ProMisE molecular subgroups across all of our evaluable cohorts.

## Rare histotypes and diversity within tumors

The role of molecular classification in rare histotypes of endometrial carcinoma has not been determined. The TCGA was restricted to cases of endometrioid and serous histology, however, the TransPORTEC cohorts and our own series included other histologies; 15% clear cell, and a combination of 6% clear cell, carcinosarcoma, undifferentiated, and mixed, in the cohorts respectively [69, 77, 79]. Fundamental features of the immunophenotype for dedifferentiated, clear cell, and mixed carcinomas have been reported [50, 54, 109–111]. Assessment of mixed tumors show that despite morphologic differences/mimicry, the majority of molecular aberrations are shared across the tumor [112]. Thus the application of ProMisE or Leiden classification systems to these cancers may be of value. Indeed in the small number of non-serous, non-endometrioid cases studied thus far, histotypes were distributed across the molecular subgroups (not confined to p53 abn subgroup). We anticipate there will be deeper characterization of unique genomic categories; e.g., dedifferentiated carcinomas within p53 wt subgroup with mutations in the SWI/SNF pathway.

Intratumoral heterogeneity in EC has been described [113, 114], and might be predicted to weaken the utility of ProMisE. However, in the cases examined, although single nucleotide variations and copy number analysis revealed some diversity between anatomic sites within an individual (at time of diagnosis) the ProMisE molecular subgroup categorization was concordant across all tumor sites (6–14 anatomic sites examined per individual) [114]. We have reported on a case of discordant ProMisE categorization between a diagnostic endometrial biopsy and final hysterectomy specimen in an individual with a dedifferentiated endometrial carcinoma [80]. This was secondary to concurrent low grade and high grade areas within the endometrium and myometrium where mismatch repair profiles differed. In rare cases, in which diverse tumor morphology is observed it may be that more than one area needs to undergo molecular testing. Certainly, gross and microscopic assessment of endometrial cancers by pathologists will need to continue just as relevant post staging data on metastases may be weighed in management. Successful integration of molecular classification will require addressing all of these issues over time, but in the interim, we anticipate a mix of current practice (histomorphologic categorization) and molecular tools for assessment of newly diagnosed ECs.

## Conclusions

We have harboured too long in a system of irreproducible categorization of endometrial carcinomas, inconsistent management within and across cancer centers, and inappropriate research investigations that grouped diverse tumors for study, making advances in research and clinical management slow or impossible in this disease site. It is essential that biologically relevant molecular features are assessed and considered for categorization of tumors, and in deciding surgical management and adjuvant therapy. This does not require abandonment of clinicopathologic parameters, many of which have been demonstrated to maintain prognostic relevance even in the post-TCGA era, but rather not to rely on them as the only or most important feature to guide management.

We have shown that in the hands of two independent research teams molecular classification of endometrial carcinomas is feasible, and identifies four prognostically distinct subgroups. Historical segregation of Type I (mostly CN low, p53 wt cases) and Type II ECs (mostly CN high, p53 abn subgroups) is inadequate and do not account for the approximately 30% of cases that are MMR-D or *POLE * EDM. All components of the molecular classifier together can be achieved at a cost* comparable to other commonly utilized clinical assays in cancer care. At minimum, this system provides objective reproducible categorization of EC’s. Familiarity with MMR and p53 IHC testing and interpretation lends to rapid adoption in any pathology department. The reproducibility of ProMisE across Canadian cancer centers is currently being evaluated.

Additional benefits of molecular classification include early identification of women who may have an inherited genetic syndrome (Lynch) who would benefit from additional screening or interventions for other Lynch-associated cancers or in whom specific therapies for their endometrial carcinomas may be more effective. For young women with EC considering delay of hysterectomy for fertility reasons (e.g., progesterone therapy), molecular classification of her diagnostic endometrial specimen could help guide management as either MMR-D (depending on germline results post hereditary cancer referral) or p53 abn categorization would discourage a conservative approach. It is still unclear how knowing the *POLE* mutation status within an individual’s EC will impact her clinical management, as favorable outcomes observed in these individuals may be either independent or secondary to increased sensitivity to DNA damaging agents (chemotherapy, radiation), and withholding treatment cannot yet be advised. Plausibly, women with p53 abn tumors with higher association of metastatic disease and aggressive clinical course would be recommended to undergo more comprehensive surgical staging and closer surveillance.  Conversely, biologically indolent tumors may be cured by simplified surgery alone and perhaps spared toxic treatment and managed by community gynecologists.

We are at an exciting juncture, but aware of the many questions still remaining (Table 1). Through clinical trials, we need to determine how molecular classification can be best integrated in to current clinical care and how will it impact outcomes. What, if any, additional parameters can better inform management? Interrogation of genotypic and phenotypic features may provide additional prognostic and predictive information. These can now be explored within the context of the four major molecular categories of tumors, and even within molecular subgroups. How reliable are IHC surrogates for mutational data in endometrial cancer? Characterization of p53 and other markers, as has been achieved in ovarian cancer [115] is needed for this disease site. Can molecular classification help interpret the natural history and direct management of cases that have historically been a great challenge to manage e.g., grade 3 endometrioid carcinoma? What is the natural history of ECs with two molecular features e.g. MMR-deficient and aberrant p53? How often are these tumors encountered and how should they be categorized? Is there a surrogate that could replace sequencing for *POLE*? Are favorable outcomes in *POLE* patients independent of treatment (e.g., can these women be spared adjuvant therapy?) Although there may be many questions to address we anticipate that molecular classification will facilitate rapid progress in research and clinical care as has been achieved through a subtype specific approach in other tumor sites.

**Table 1:**
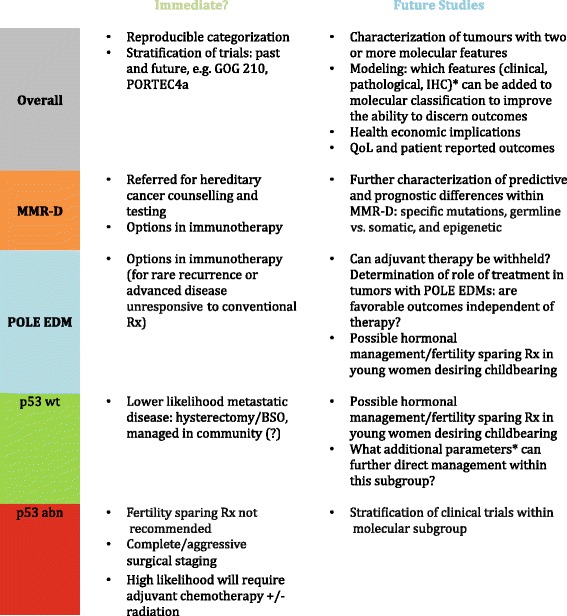
Potential changes in practice through molecular categorization

*Features that have been historically used in risk classification or are considered prognostic markers in other series

In summary, whilst the combination of histomorphology and clinical factors has proven to be insufficiently reproducible, prognostic and predictive, two molecular classifiers based on the TCGA study show great potential as pragmatic and effective tools to stratify patient risk and subsequent care decisions. Given the high and increasing incidence of endometrial cancer and the societal cost of over- and under-treatment there is urgent need for prospective clinical studies to determine how best to utilize these tools.

*For ProMisE; the materials, assay and interpretation costs total < $300 USD

## Abbreviations

EC: Endometrial cancer
TCGA: The cancer genome atlas
ESMO: European Society for Medical Oncology
LVSI: Lymph-vascular space invasion
*POLE* EDM: Polymerase epsilon exonuclease domain mutation
MMR: Mismatch repair
MMR-D: Mismatch repair deficiency
